# Middle meningeal artery embolization for organized chronic subdural hematoma combined with minimal evacuation surgery: Two case reports

**DOI:** 10.1016/j.radcr.2024.09.062

**Published:** 2024-09-25

**Authors:** Yoshinori Kadono, Ryuichiro Kajikawa, Takashi Tsuzuki, Haruhiko Kishima

**Affiliations:** a Department of Neurosurgery, Takatsuki General Hospital, Takatsuki, Osaka, Japan; b Department of Neurosurgery, Sakai City Medical Center, Sakai, Osaka, Japan; c Department of Neurosurgery, Osaka University Graduate School of Medicine, Suita, Osaka, Japan

**Keywords:** Organized chronic subdural hematoma, Middle meningeal artery embolization, Thrombocytopenia, Intervention radiology

## Abstract

Organized chronic subdural hematoma (OCSDH) is a rare condition lacking standardized treatment protocols. Middle meningeal artery (MMA) embolization has recently demonstrated promising outcomes in managing chronic subdural hematoma (CSDH). We present 2 cases of OCSDH treated with endovascular embolization and minimal evacuation surgery. The first case involved an 83-year-old male with a history of left CSDH drainage, admitted urgently due to right hemiplegia and dysarthria. CT scans confirmed recurrent CSDH. A small craniotomy was performed to decompress the thick hematoma, followed by drain placement. Postoperative magnetic resonance imaging (MRI) indicated OCSDH. Seven days later, MMA embolization with 25% n-butyl-2-cyanoacrylate (NBCA) was performed under local anesthesia. The patient's symptoms improved, and the hematoma resolved within 6 months without recurrence. The second case involved a 76-year-old male with right CSDH and thrombocytopenia (platelet count of 19,000/µL), diagnosed with immune thrombocytopenia. MRI indicated OCSDH. Due to the risk associated with craniotomy, a burr hole perforation and MMA embolization were planned, accompanied by a platelet transfusion. Left MMA embolization with 20% NBCA was performed, followed by burr hole enlargement for decompression and drain placement. The patient's symptoms improved postoperatively, and his platelet count stabilized with steroid therapy and thrombopoietin. The hematoma resolved within 3 months without recurrence. These cases indicate that MMA embolization combined with small craniotomy or perforation may be an effective treatment strategy for OCSDH.

## Introduction

Chronic subdural hematoma (CSDH) is a common condition resulting from head trauma. However, a subset of these hematomas, only 0.5%-2.0% of all CSDHs, progresses into a more complex disease known as organized chronic subdural hematoma (OCSDH). This condition is characterized by a thick, vascularized membrane encapsulating the hematoma [1,2]. This intricate structure contributes to the persistence and resistance to resorption of the hematoma, often necessitating therapeutic intervention. While the exact pathophysiology remains unclear, factors such as inflammation, angiogenesis, and coagulopathy are believed to play important roles in developing OCSDH [[2], [3], [4]].

Despite extensive research, the optimal management of OCSDH remains a clinical challenge. Traditionally, surgical intervention, including a large craniotomy with hematoma removal and membrectomy, has been the mainstay of treatment [1,[5], [6], [7]]. This invasive approach carries significant risks, particularly in older people. Recent advancements in endovascular techniques have offered a promising alternative. Middle meningeal artery embolization (MMAE) has emerged as a promising minimally invasive procedure for CSDH treatment [8], with some studies suggesting it may also be effective for OCSDH [[9], [10], [11], [12]]. Despite these advantages, endovascular methods are less commonly performed compared to surgical interventions, largely due to limited experience, variability in procedural success, and the need for specialized equipment and expertise. However, recent case reports have demonstrated the effectiveness of MMAE in treating OCSDH, offering a promising alternative for patients who are at high risk for traditional surgery [[9], [10], [11], [12]]. This report presents 2 cases of OCSDH managed with MMAE combined with perforation surgery or small craniotomy, highlighting the evolving role of endovascular techniques in treating this challenging condition.

## Case description

**Fig. 1:**
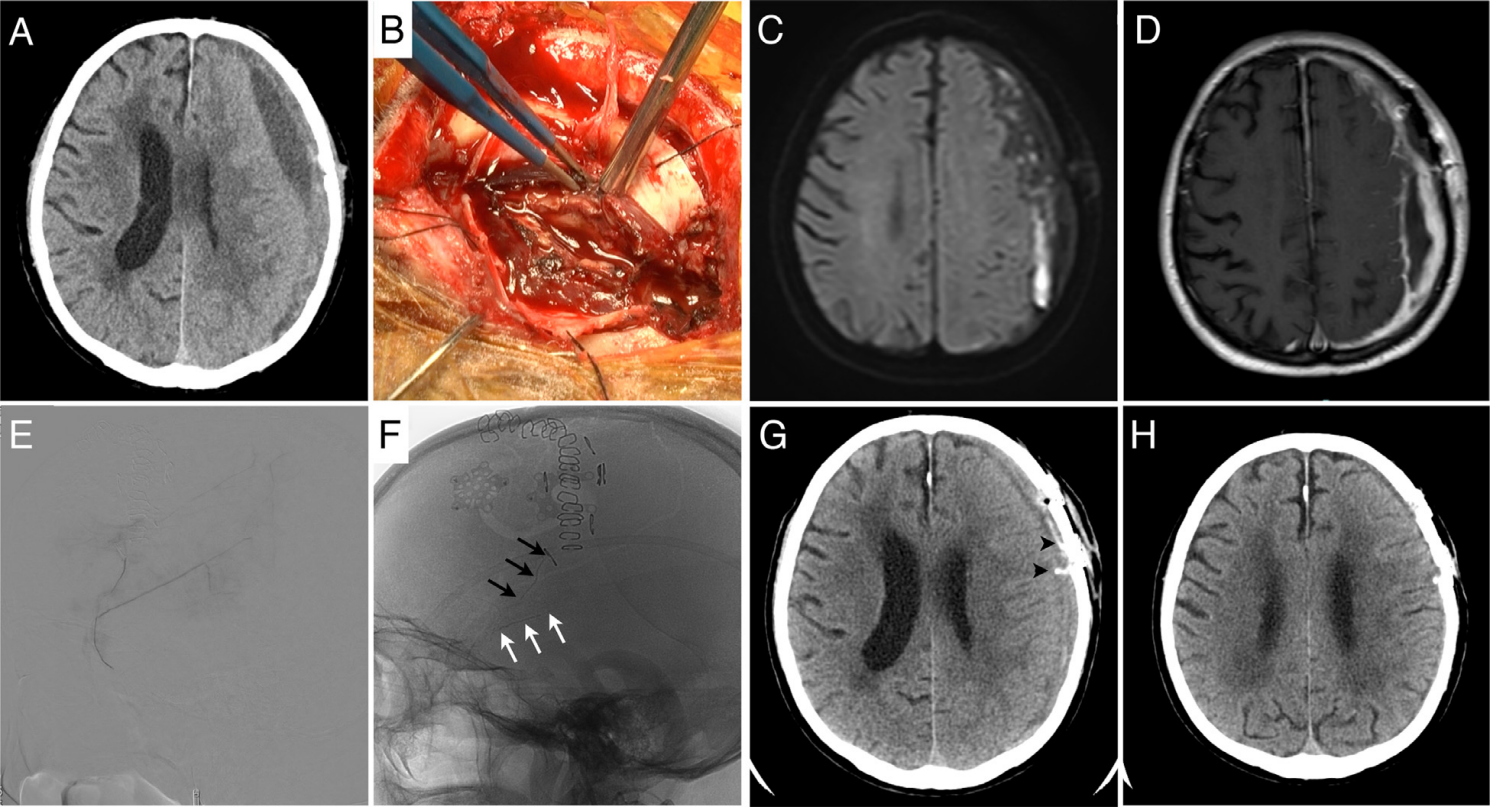
An 83-year-old male with a history of left chronic subdural hematoma drainage presented with right hemiplegia. (A) A head computed tomography (CT) scan on admission showed a mixed-density left subdural hematoma with midline shift. (B) A small craniotomy and dura incision revealed a solid hematoma, and the outer thick membrane was removed as much as possible. (C, D) Diffusion-weighted images and gadolinium-enhanced T1-weighted images showed the solid vascularized membrane of an organized chronic subdural hematoma (OCSDH). (E) A lateral view of an angiogram of the left middle meningeal artery showed the vascular foci feeding the hematoma. (F) The left middle meningeal artery embolization was performed with 25% n-butyl-2-cyanoacrylate (NBCA) for the frontal branch (black arrows) and parietal branch (white arrows). (G) A postoperative CT scan showed the injected NBCA in the inner and outer membrane of OCSDH (arrowhead). (H) A CT scan revealed that the hematoma had resolved, with no recurrence in 6 months.

**Case 1:** An 83-year-old male with a history of left CSDH drainage 1 and a half months ago presented with right upper limb paralysis and dysarthria. A head computed tomography (CT) scan revealed a recurrence of left subdural hematoma with midline shift (Fig. 1A). He was urgently admitted with a diagnosis of recurrent CSDH, and the following morning, a perforation surgery via the previous burr hole was attempted. During surgery, the outer membrane of the hematoma was thick and solid, making evacuation via the burr hole unsuccessful. Therefore, a small craniotomy was performed to decompress the solid hematoma and outer membrane as much as possible (Fig. 1B), and a drain was placed into the cavity. Postoperative magnetic resonance imaging (MRI) revealed a thick enhanced membrane, indicating it was an OSCDH (Fig. 1, Fig. 1). Seven days later, the patient underwent MMAE under local anesthesia. A 1.5-French flow-controlled microcatheter was navigated to the left middle meningeal artery, and MMAE was performed with 25% n-butyl-2-cyanoacrylate (NBCA) for the frontal branch and parietal branch (Fig. 1, Fig. 1). A postoperative CT scan showed that the NBCA was injected into the thick outer membrane (Fig. 1G). The patient's symptoms improved, and he was discharged on the 13th postoperative day. The hematoma resolved in 3 months, with no recurrence in 6 months (Fig. 1H).

**Fig. 2:**
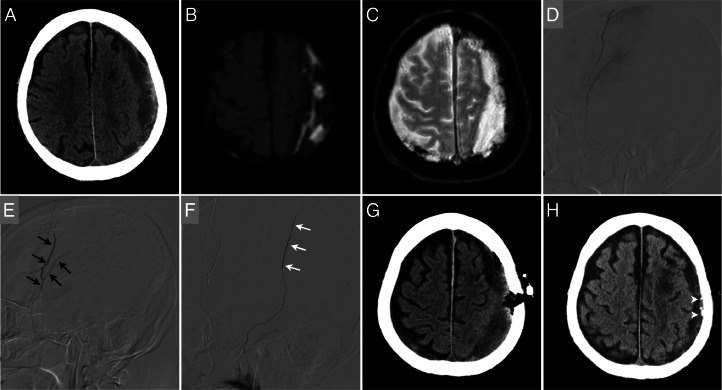
A 76-year-old male with right hemiplegia and thrombocytopenia was admitted. (A) A head computed tomography (CT) scan on admission showed a left subdural hematoma with a tough membrane and a slight midline shift. (B, C) Diffusion-weighted images and T2-star weighted images indicated the solid membrane of an organized chronic subdural hematoma. (D) A lateral view of an angiogram of the left middle meningeal artery showed the feeding arteries of the hematoma. (E, F) The left middle meningeal artery embolization was performed with heated 20% n-butyl-2-cyanoacrylate (NBCA) for the frontal branch (black arrows) and parietal branch (white arrows), followed by burr hole surgery after platelet transfusion. (G) A postoperative CT scan showed partial removal of the subdural hematoma via a burr hole. (H) After medication for immune thrombocytopenia, the hematoma resolved, and the injected NBCA was in the scar of the outer membrane (arrowhead) in 3 months.

**Case 2**: A 76-year-old male with right hemiplegia and headache was transferred to our hospital. CT scans revealed a left CSDH with a slight midline shift (Fig. 2A), and a blood test showed thrombocytopenia with a platelet count of 19,000/µL upon admission. Until the cause of the thrombocytopenia was identified, his right hemiplegia gradually progressed, and his platelet count dropped to 1000/µL despite repeated platelet transfusions. A bone marrow biopsy revealed a diagnosis of immune thrombocytopenia. An MRI scan suggested the hematoma was an OCSDH with a thick membrane (Fig. 2, Fig. 2). Considering the risk of craniotomy under thrombocytopenia, a perforation surgery and MMAE were planned following a Human Leukocyte Antigens (HLA) -matched platelet transfusion. On the 14th hospital day, under local anesthesia, MMAE was performed, and subsequent burr hole surgery involved hematoma evacuation and drain placement after a 20-unit HLA-matched platelet transfusion. A 2.7-French microcatheter with a 0.014-inch microcatheter was inserted into the left middle meningeal artery through a 4-French intermediate catheter. An angiogram of the left middle meningeal artery showed the branches feeding the OCSDH (Fig. 2D), and heated 20% NBCA was injected into the frontal and parietal branches (Fig. 2, Fig. 2). A postoperative CT scan illustrated the partial removal of the OCSDH via a burr hole and the injected NBCA in the thick outer membrane (Fig. 2G). After the treatment, he started steroid therapy and thrombopoietin administration. His neurological symptoms improved day by day, and his platelet count stabilized. He was discharged home on the 30th day with no recurrence at the 3-month follow-up (Fig. 2H).

## Discussion

OCSDH represents a complex progression of CSDH, characterized by a thick, vascularized membrane surrounding the hematoma. This neovascularization and membrane formation contribute to the persistence and resistance of the hematoma. Factors such as inflammation, angiogenesis, and coagulopathy are believed to play key roles in the pathogenesis of recurrent CSDH and OSCDH [2,3]. As a result, managing OCSDH poses significant challenges, particularly because the condition often requires more invasive therapeutic interventions than standard CSDH. On the other hand, MMAE has recently gained attention as a less invasive treatment for recurrent or refractory CSDH [[13], [14], [15]]. Mandai et al. [8] first reported this technique, which involves blocking the blood flow through the middle meningeal artery that supplies the hematoma membrane. By reducing the blood supply, MMAE helps to shrink the hematoma and prevent its recurrence. Previous studies report a recurrence rate of 8%–30% with burr hole surgery alone, compared to 2.7%-5.4% with the addition of MMAE [16]. In addition to the lower recurrence rate, the benefits of additional MMAE include reduced intraoperative blood loss and shorter hospital stays. These advantages are particularly relevant for elderly patients or those with comorbidities and antithrombotic therapy who are at high risk of recurrence. Moreover, from its previously mentioned pathophysiological perspective, MMAE is reasonable as a curative treatment for CSDH. Therefore, this novel technique could be helpful for patients with OSCDH with a thick, neovascularized membrane.

Traditional management of OCSDH typically involves a large craniotomy with hematoma removal and durotomy [[5], [6], [7]]. While aggressive surgery involving total removal of the abnormal proliferative membrane and hematoma is effective, this approach is highly invasive. It carries significant risks, including infection, bleeding, general anesthesia and prolonged recovery, particularly in elderly patients or those with comorbidities. Our cases demonstrate the efficacy of combining MMAE with a less invasive surgical technique, such as small craniotomy or burr hole surgery. This novel approach reduces the extent of surgery required, thereby minimizing the associated risks and improving recovery times. The dual approach of MMAE and minor surgery represents a novel minimally invasive treatment for OCSDH, combining reducing the hematoma's blood supply with less invasive hematoma evacuation. This study is one of the first to report successful outcomes using this combined less invasive method for OCSDH, highlighting its potential as a viable alternative to traditional surgical management.

While our results were positive, larger studies are needed to confirm the efficacy and safety of combining MMAE with minimally invasive surgery for OCSDH. Some case reports of OCSDH treated with MMAE and traditional craniotomy have been published previously [9,11,12]. However, Yokoya et al. recently reported 2 cases of OCSDH treated with MMAE and minor craniotomy surgery, similar to our cases [10]. This approach could be particularly beneficial for patients at high risk from large craniotomy or general anesthesia. Although OCSDH is a rare disease, future research should focus on comparing outcomes between this combined less invasive approach and traditional large craniotomy with MMAE. Additionally, exploring using different embolic agents or supplementary therapies, such as endoscopic surgery, could further optimize outcomes [17]. Developing standardized protocols for the management of OCSDH with MMAE might significantly advance clinical practice and improve patient care.

## Conclusion

These cases illustrate that MMAE combined with small craniotomy or burr hole surgery may be an effective treatment for OCSDH, particularly in patients at high risk for craniotomy. This combined approach may provide a minimally invasive and safe treatment option for OCSDH, leading to improved patient outcomes. Future studies with larger cohorts are essential to validate these findings and optimize the treatment protocol for OCSDH.

## Declaration of generative AI and AI-assisted technologies in the writing process

The authors declare the limited usage of artificial intelligence (AI)-assisted technology for English editing and confirm that no images were manipulated using AI. After using this tool, the authors reviewed and edited the content as needed and take full responsibility for the content of the publication.

## Patient consent

We declare that the patients’ written consent for this study as our appropriate form were obtained.
